# An Interesting Case of Disseminated Histoplasmosis in a Patient With Hemophagocytic Lymphohistiocytosis

**DOI:** 10.7759/cureus.36404

**Published:** 2023-03-20

**Authors:** Rachel Ann Pellegrino, Roopa Naik, Atul Bali

**Affiliations:** 1 Internal Medicine, Kansas City University, Danville, USA; 2 Medicine, Geisinger Commonwealth School of Medicine, Scranton, USA; 3 Internal Medicine/Hospital Medicine, Geisinger Health System, Wilkes-Barre, USA; 4 Internal Medicine/Nephrology, Geisinger Medical Center, Danville, USA; 5 Internal Medicine/Nephrology, Geisinger Health System, Wilkes-Barre, USA

**Keywords:** opportunistic fungal infection, complication of rheumatoid arthritis, rheumatoid arthritis, ra, infectious disease pathology, histoplasmosis in bone marrow

## Abstract

Histoplasmosisis a common mycosis in North and Central America caused by *Histoplasma capsulatum*. Affected patients typically remain asymptomatic. However, in some individuals, histoplasmosis can present as a severe illness, usually occurring in patients with underlying risk factors such as the immunocompromised (e.g., acquired immunodeficiency syndrome (AIDS), treatment with immunosuppressive agents), and the elderly without underlying immunocompromised conditions. Here, we present a case of disseminated histoplasmosis diagnosed as an incidental finding on bone marrow biopsy while treating a hospitalized patient for hemophagocytic lymphohistiocytosis. The patient presented with weight loss, anorexia, fatigue, and generalized weakness. The patient was successfully treated with amphotericin B and eventually transitioned to itraconazole.

## Introduction

*Histoplasma capsulatum* is a dimorphic fungus typically asymptomatic in immunocompetent hosts [1]. Endemic to Midwestern states in the Ohio and Mississippi River valleys, histoplasmosis can be found worldwide but predominantly in North and Central America [2]. The infection is transmitted by the inhalation of spores in soils that have been contaminated by bat or bird droppings [1]. Although severe illness caused by *Histoplasma capsulatum *is rare, disseminated infections in immunocompromised hosts, such as those with acquired immunodeficiency syndrome (AIDS) or of extremes of age, have been reported in the literature [2]. Here, we report a case of disseminated histoplasmosis incidentally found in a patient with a history of rheumatoid arthritis who presented with weight loss and weakness, diagnosed with hemophagocytic lymphohistiocytosis during the same hospitalization.

## Case presentation

A 63-year-old Caucasian female with a history of rheumatoid arthritis (RA) and depression presented to our hospital for two months of cough, lethargy, weakness, and weight loss. She had lost about 50 pounds over the past year, but her condition had deteriorated rapidly in the preceding two months. She was on weekly methotrexate and twice daily hydroxychloroquine before her presentation. On a prior routine visit with her primary care physician preceding her weight loss, no overt concerning findings were noted. On presentation, she had a fever of 102.8°F, a heart rate of 117 beats per minute, and a respiratory rate of 28 per minute. Her body mass index (BMI) at admission was 13.79 (BMI 18.5 - 24.9). Initial laboratory assessments are listed in Table 1.

**Table 1:** Laboratory values on the day of admission

Laboratory parameters		Lab values		Reference values	
White blood cell count		5.06/µL		4.00–11.00/µL	
Hemoglobin		8.2 g/dL		12.0-15.0 g/dL	
Platelet count		85,000/µL		140,000–400,000/µL	
Aspartate transaminase		43 IU/L		15–37 IU/L	
Alanine transaminase		20 IU/L		0–56 IU/L	
Total bilirubin		0.7 mg/dL		0.2–1.3 mg/dL	
Serum creatinine		1.20 mg/dL		0.6–1.3 mg/dL	
Phosphorus		1.7 mg/dL		2.8 to 4.5 mg/dL	
Potassium		3.4 mmol/L		3.6 to 5.2 mmol/L	
Magnesium		1.3 mh/dL		1.7 to 2.2 mg/dL	
Lactate dehydrogenase		479 U/L		125-243 U/L	
Ferritin		5928 ng/mL		8-252 ng/mL	
International normalized ratio (INR)		1.8		1.0-2.0	
Iron		29 ug/dL		40-145 ug/dL	
Fibrinogen		37 mg/dL		200-400 mg/dL	
Iron		29 ug/dL		40-145 ug/dL	
Total iron-binding capacity		275 ug/dL		250-450 ug/dL	
Urinalysis					
White blood cell count		17/high power field		0–3/high power field	
Red blood cell count		1 / high power field		0–3/high power field	
Leukocyte esterase		Small		Absent	
Nitrites		Negative		Absent	
Bacteria		Many		Absent	

**Figure 1:**
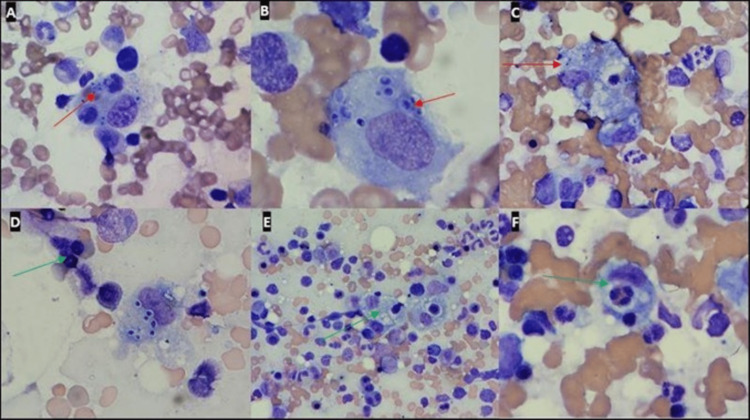
Bone marrow histopathology images with Histoplasma and hemophagocytosis *Histoplasma* (A-C) and hemophagocytosis (C-F) in the patient’s marrow smears (Wright Giemsa, 1000x oil immersion). Numerous small, narrow-base budding yeast cells within macrophages (red arrows in A-C). Hemophagocytes with ingested granulocytes (green arrow in D-F).

Computed tomography (CT) of the chest showed no evidence of nodules, infiltrates, or any other abnormality. In contrast, CT of the abdomen and pelvis showed a non-distended gallbladder with likely surrounding fluid without any other abnormalities and was negative for lymphadenopathy or hepatosplenomegaly. Due to bacteriuria in the context of fever, a provisional diagnosis of urinary tract infection was made, and the patient was started on intravenous ceftriaxone. Subsequently, urine cultures grew pan-sensitive Escherichia coli, while blood cultures showed no growth. The viral hepatitis panel and human immunodeficiency virus (HIV) panel were negative. Hematology was consulted for the evaluation of pancytopenia. Peripheral smear showed normocytic anemia, neutrophilia with left shift, absolute lymphopenia, and moderate thrombocytopenia. Over the next few days, the patient received several units of packed red blood cells, platelets, and fresh frozen plasma due to worsening pancytopenia. She also developed painful ulcerations involving her lips and tongue and was started on an acyclovir suspension for possible herpes simplex infection. Bone marrow biopsy revealed hemophagocytosis, hypercellular marrow (60-70%) with erythroid hyperplasia and dyspoiesis, increased bone marrow storage of iron, and the unexpected finding of numerous small, small, narrow-based budding yeast cells noted intracellularly within macrophages (Figure 1).

A diagnosis of hemophagocytic lymphohistiocytosis (HLH) was made, with the concomitant finding of disseminated histoplasmosis (DH). HLH diagnosis criteria are enlisted in Table 2 [3].

**Table 2:** Hemophagocytic lymphohistiocytosis diagnosis criteria Five of the above criteria are needed to establish the diagnosis of hemophagocytic lymphohistiocytosis.

Hemophagocytic lymphohistiocytosis diagnostic criteria
Fever ≥38.5°C
Splenomegaly
Peripheral blood cytopenia
Plus at least 2 of the following
Hemoglobin <9 g/dL (for infants <4 weeks, hemoglobin <10 g/dL)
Platelets <100,000/microL
Hypertriglyceridemia (fasting triglycerides >265 mg/dL)
Absolute neutrophil count <1000/microL
Hypofibrinogenemia (fibrinogen <150 mg/dL)
Hemophagocytosis in bone marrow, spleen, lymph node, or liver
Low or absent nature killer (NK) cell activity
Ferritin >500 ng/mL
Elevated soluble CD25 (soluble IL-2 receptor alpha (sIL-2R))

Due to the risks associated with a delay in diagnosis, some experts deem a modified criterion sufficient to diagnose HLH: three of four clinical findings (fever, splenomegaly, cytopenias, hepatitis) plus abnormality of one of four immune markers (hemophagocytosis, increased ferritin, hypofibrinogenemia, absent or very decreased nature killer (NK) cell function) [3]. Since our patient presented with fever, had low fibrinogen, elevated ferritin, with evidence of hemophagocytosis, cytopenias, and transient hepatitis, this constellation of findings led to the diagnosis of HLH in our patient. Her preceding pulmonary symptoms and weight loss, mucocutaneous ulcerations, with a subsequent note of *Histoplasma capsulatum *(H. capsulatum) on bone marrow biopsy, suggested DH was the likely etiology of this subacute presentation. The differential diagnosis for this patient is broad, including infectious, noninfectious, and cardiac causes [4-15].

The patient was started on high-dose intravenous dexamethasone. As the patient responded well and the cell counts improved on steroid therapy, etoposide was held and was not used. Since the diagnosis of DH was made, antibacterial agents were stopped in favor of starting intravenous amphotericin B, followed by the addition of oral itraconazole to her regimen. Given her baseline characteristics suggesting chronic susceptibility to infections (due to rheumatoid arthritis and the need for immunosuppressive therapy), maintenance of long-term itraconazole was recommended. While the patient had a prolonged hospital course, complicated by renal manifestations of amphotericin B and subsequent development of refeeding syndrome, overall, her clinical course improved with a slow but reassuring response to treatment.

Ultimately, she was discharged to a skilled nursing facility with outpatient follow-up with an infectious disease specialist, hematologist, and primary care physician. An outpatient monitoring plan was devised to evaluate her response to itraconazole by periodically monitoring her urine and serum for Histoplasma and Blastomyces antigens and monthly lab assessments of her hematological and metabolic parameters.

## Discussion

*Histoplasma capsulatum* is a fungus that grows as a mold in the environment. Once the spores are inhaled into the lungs, they transform into their yeast form, which can also occur in environments over 37ºC. Akin to tuberculosis, the neutrophils, macrophages, lymphocytes, and natural killer cells respond to fight the infection. Macrophages also have an inherent fungicidal capacity. Most human hosts who inhale the spores remain asymptomatic. However, the disease can disseminate in those with macrophage activation deficiencies [16]. Similarly, individuals receiving immunosuppressive medications that alter their immune response are susceptible. On bone marrow biopsy, this can be seen as macrophages containing 2-to-4-micron yeast structures [17].

Disseminated histoplasmosis typically presents in individuals as pulmonary histoplasmosis [2]. Data from two large urban outbreaks in Indianapolis suggested that immunosuppression, older age, and coexisting chronic illnesses were the primary risk factors for acquiring disseminated histoplasmosis [18]. It is well-known that an immunocompromised state and old age predispose to disseminated infections [19,20]. Of the 66 cases of disseminated histoplasmosis reviewed in the above study, two-thirds of patients reported some combination of central nervous system findings, splenomegaly, hepatomegaly, and lymphopenia [18].

Mucosal lesions have been noted in the literature in HIV-infected patients with disseminated histoplasmosis [21], even though the differential diagnosis of mucosal lesions in HIV patients can be broad [22]. A study in Brazil of 36 HIV-infected patients reported that 33 had mucocutaneous histoplasmosis (oral papules, crusted papules, and oro-mucosal erosions) as a manifestation of disseminated disease [18]. However, this is not commonly reported in individuals without HIV. HLH is a syndrome of excessive inflammation and tissue destruction typically caused by excessive immune activation [17]. Seen predominantly in infants from birth to 18 months of age, it has less commonly been noted in older adults. Both infection and genetic predispositions may contribute to disease etiology, but its pathophysiology is poorly understood. Preexisting autoimmune and rheumatologic diseases in adults are reported in cases of HLH in the literature [23-27]. It can also be associated with malignancies like Hodgkin lymphoma [28].

There is limited literature on the superimposition of HLH on disseminated histoplasmosis. However, the treatment for patients acutely ill with HLH is chemotherapy and immunotherapy, followed by bone marrow transplant in relapsing or recurrent disease [29]. Conversely, chemotherapy and immunotherapy can lead to immunosuppression, predisposing to opportunistic infections with organisms such as *Histoplasma capsulatum*.

The appropriate treatment of severely ill patients with disseminated histoplasmosis requiring hospitalization is a lipid formulation of amphotericin B followed by oral itraconazole for maintenance therapy [30]. These patients typically respond well to amphotericin B if they can tolerate the side effects and stop taking the formulation after one to two weeks of treatment [30]. Itraconazole is utilized for maintenance therapy for at least a year or as long as the patient remains immunosuppressed, which may be for a lifetime for most patients with disseminated histoplasmosis [31]. Patients with mild to moderate disseminated disease are treated solely with itraconazole [30]. A study found that amphotericin B was effective for severe cases of disseminated histoplasmosis when a cumulative dose of at least 500 mg was administered [18].

## Conclusions

There are several unique features in our reported case. As mentioned, our patient had a history of rheumatoid arthritis, a predisposing factor to disseminated histoplasmosis. One of her presenting symptoms was a mucocutaneous manifestation, which has only previously been noted in HIV-infected patients. Our patient’s unique presentation and concurrent diagnosis of HLH have not been cited in the literature, thus resulting in a complicated approach to treatment. Mortality rates of HLH in immunosuppressed adults are high when prompt treatment is not instituted, as is the case with disseminated histoplasmosis. Our patient had underlying RA, which might have led to an immunocompromised state, resulting in infection with *Histoplasma capsulatum* with subsequent dissemination. It is plausible that the macrophage activation in response to Histoplasma infection and its subsequent dissemination led to a surge in cytokine release that, in our susceptible patient, served as the trigger for HLH. To our knowledge, this is the first reported case in the literature of a patient with disseminated histoplasmosis and associated HLH, and thus sharing this unique case would help enhance our understanding of both of these elusive disease processes.
